# A Regulatory *MDM4* Genetic Variant Locating in the Binding Sequence of Multiple MicroRNAs Contributes to Susceptibility of Small Cell Lung Cancer

**DOI:** 10.1371/journal.pone.0135647

**Published:** 2015-08-14

**Authors:** Feng Gao, Xiangyu Xiong, Wenting Pan, Xinyu Yang, Changchun Zhou, Qipeng Yuan, Liqing Zhou, Ming Yang

**Affiliations:** 1 Health Division of Guard Bureau, General Staff Department of Chinese PLA, Beijing, China; 2 College of Life Science and Technology, Beijing University of Chemical Technology, Beijing, China; 3 Clinical Laboratory, Shandong Cancer Hospital, Shandong Academy of Medical Sciences, Jinan, Shandong Province, China; 4 Department of Radiation Oncology, Huaian No. 2 Hospital, Huaian, Jiangsu Province, China; Medical College of Soochow University, CHINA

## Abstract

A functional rs4245739 A>C single nucleotide polymorphism (SNP) locating in the *MDM4*3’-untranslated (3’-UTR) region creates a miR-191-5p or miR-887-3p targeting sites. This change results in decreased expression of oncogene *MDM4*. Therefore, we examined the association between this SNP and small cell lung cancer (SCLC) risk as well as its regulatory function in SCLC cells. Genotypes were determined in two independent case-control sets consisted of 520SCLC cases and 1040 controls from two regions of China. Odds ratios (ORs) and 95% confidence intervals (CIs) were estimated by logistic regression. The impact of the rs4245739 SNP on miR-191-5p/miR-887-3p mediated *MDM4* expression regulation was investigated using luciferase reporter gene assays. We found that the *MDM4* rs4245739AC and CC genotypes were significantly associated with decreased SCLC susceptibility compared with the AA genotype in both case-control sets (Shandong set: OR = 0.53, 95% CI = 0.32–0.89, *P* = 0.014; Jiangsu set: OR = 0.47, 95% CI = 0.26–0.879, *P* = 0.017). Stratified analyses indicated that there was a significantly multiplicative interaction between rs4245739 and smoking (*P*_interactioin_ = 0.048). After co-tranfection of miRNAs and different allelic-*MDM4* reporter constructs into SCLC cells, we found that the both miR-191-5p and miR-887-3p can lead to significantly decreased *MDM4* expression activities in the construct with C-allelic 3’-UTR but not A-allelic 3’-UTR, suggesting a consistent genotype-phenotype correlation. Our data illuminate that the *MDM4*rs4245739SNP contributes to SCLC risk and support the notion that gene 3’-UTR genetic variants, impacting miRNA-binding, might modify SCLC susceptibility.

## Introduction

Lung cancer is the leading causes of cancer death in the world, with more than1,000,000 deaths per year [1]. Lung cancer is commonly classified as small-cell lung cancer(SCLC) and non-small-cell lung cancer (NSCLC). Though accounting for only approximately twenty percent of all lung cancer cases, SCLC is much more aggressive compared to NSCLC [2,3]. As a malignancy with poor prognosis, SCLC shows high growth fraction, rapid doubling time, and early development of widespread metastases [2,3]. When diagnosed, most SCLC patients have metastasis and bad prognosis if left untreated [3]. Accumulated epidemiological evidences indicate that heavy tobacco smoking is a main environmental risk-factor of this disease [2,3]. Recent progresses on genome-wide association studies (GWAS) have revealed that multiple novel genetic variations are associated with lung cancer susceptibility [4–12]. However, most studies examined both lung cancer subtypes or only focused on NSCLC. Interestingly, the majority of identified risk loci do not significantly influence the risk of lung cancer differentially by histology, indicating that there might be different genetic makeup impacts risk of SCLC or NSCLC [4–12]. Therefore, discovery of novel SCLC-risk-associated SNPs might be a potentially valuable path towards illuminating etiology of SCLC.

MDM4, also known as MDMX or HDMX, is a structurally homologous protein of MDM2. MDM4 shares an NH_2_-terminal P53-binding domain with MDM2 and can inhibit activities of P53 in various malignancies [13–17]. As the guardian of human genome, P53 functions at the center of a complex molecular network to mediate tumor suppression. As a well-known oncogene, overexpressed MDM4 in human tumors including lung cancer led to depressed P53 activities and, thus, tumorigenesis. In line with this, mouse model with overexpressed MDM4 via transgenetic technology showed spontaneous carcinogenesis during their lifespan [18].

There is an *MDM4*3’-untranslatedregion (3’-UTR) rs4245739A>C SNP locating in the target binding site for two miRNAs (miR-191-5p and miR-887-3p) [19,20]. miR-191-5p and miR-887-3p could selectively bind to *MDM4*3’-UTR with the rs4245739C allele but not 3’-UTR with the A allele. This allelic miRNAs’ binding leads to elevated expression levels of *MDM4* mRNA and/or protein among rs4245739A allele carriers with cancers [19–22]. Two GWAS showed that the *MDM4* rs4245739 A-allele is significantly associated with increased risk of both prostate cancer and breast cancer [23,24]. Several case-control studies using candidate gene strategy also confirmed the positive association between this polymorphism and susceptibility of ovarian cancer, breast cancer, esophageal squamous cell carcinoma and non-Hodgkin lymphoma in different ethnic populations [20,22,25,26]. However, the involvement of this functional SNP in SCLC is still largely unknown. Considering the essential role of MDM4 in carcinogenesis, we hypothesized that the *MDM4*rs4245739 SNP may contribute to SCLC susceptibility via allelic regulation ofmiR-191-5p and/or miR-887-3p binding affinity and, thus, MDM4 expression. In the current study, we conducted a two-stage case-control study of SCLC recruited from Jinan city (Shandong Province, China) and Huaian city (Jiangsu Province, China). Furthermore, to validate the biological function of this polymorphism, we investigated the allelic regulation of miR-191-5p and miR-887-3p on MDM4 in SCLC cells.

## Materials and Methods

### Study subjects

This study consisted of two case-control sets: (i) Shandong case-control set: 320 SCLC patients from Shandong Cancer Hospital, Shandong Academy of Medical Sciences (Jinan, Shandong Province, China) and sex- and age-matched (±5 years) 640 controls. Patients were recruited between June 2009 and November 2014 at Shandong Cancer Hospital. Control subjects were randomly selected from a pool of 4500 individuals from a community cancer-screening program for early detection of cancer conducted in Jinan city as described in detail previously [22]. (ii) Jiangsu case-control set: 200 SCLC patients from Huaian No. 2 Hospital (Huaian, Jiangsu Province, China) and sex- and age-matched 400 controls. Patients were consecutively recruited between January 2009 and January 2015 at Huaian No. 2 Hospital. Controls were cancer-free individuals selected from a community cancer-screening program (3600 individuals) for early detection of cancer conducted in Huaian city as described in detail previously [22]. Individuals who smoked one cigarette per day for over 1 year were considered as smokers [22]. All subjects were unrelated ethnic Han Chinese. This study was approved by the Institutional Review Boards of Huaian No. 2 Hospital and Shandong Cancer Hospital, Shandong Academy of Medical Sciences. Written informed consent was obtained from each subject at recruitment.

### SNP genotyping

PCR-based restriction fragment length polymorphism (RFLP) was utilized to determine *MDM4* rs4245739A>C genotypes as described in detail previously [22,25,26]. In brief, the primers used for amplifying DNA segments with the rs4245739 site (the mismatch base is underlined) were 5′-AAGACTAAAGAAGGCTGGGG-3′ and 5′-TTCAAATAATGTGGTAAGTGACC-3′. Restriction enzyme *Msp*I (New England Biolabs) was used to digest the PCR products and distinguish the rs4245739 genotypes. A 10% random sample was reciprocally examined by different person, and the reproducibility was 98.7%. Additionally, a 5% random samples were also tested via Sanger sequencing, and the reproducibility was 100%.

### Plasmid construction

The *MDM4* rs4245739A and rs4245739C allelic reporter constructs were prepared by amplifying part of the *MDM4*3’-UTR region from subjects homozygous for the rs4245739AA or rs4245739CC genotype. The PCR primers used were as follows: 5′-AACTCTAGAGGTAGTACGAACATAAAAATGC-3′ and 5′-AACTCTAGACTGCATAAAGTAATCCATGG-3′, which includes the *Xba* I restriction site (underlined sequences). The PCR products were digested with *Xba* I (New England Biolabs) and ligated, respectively, into an appropriately digested pGL3-control vector (Promega). The constructs were designated aspGL3-rs4245739A and pGL3b-rs4245739C, respectively. The inserts were confirmed by DNA sequencing.

### Dual luciferase reporter gene assay

A firefly luciferase reporter plasmid (pGL3-control, pGL3-rs4245739A or pGL3-rs4245739C), a renilla luciferase vector (pRL-SV40, Promega) plus 30 nMsmall RNAs (miR-191-5p mimics, miR-887-3p mimics or negative control RNAs) (Genepharma, Guangzhou, China) were co-transfected into SCLCH446 cells with Lipofectamine 2000 (Invitrogen, Carlsbad, CA). Luciferase activity was determined at 48h after transfection using a luciferase assay system (Promega) as previously described [27–29]. Three independent transfection experiments were performed and each was done in triplicate. Fold increase was calculated by defining the activity of empty pGL3-control vector as 1.

### Statistical analyses

As described in detail previously [22, 30–32], the differences in demographic variables and genotype distributions of *MDM4* rs4245739 SNP between SCLC cases and controls were calculated using Pearson’s χ^2^ test. Associations between *MDM4* rs4245739 genotypes and SCLC risk were estimated by odds ratio (OR) and their 95% confidence intervals (CIs) computed using unconditional logistic regression model. All ORs were adjusted for age, sex and smoking status, where it was appropriate. We tested the null hypotheses of multiplicative gene-covariate interaction and evaluated departures from multiplicative interaction models by including main effect variables and their product terms in the logistic regression model [30–32]. A *P* value of less than 0.05 was used as the criterion of statistical significance, and all statistical tests were two-sided. All analyses were performed with SPSS software package (Version 16.0, SPSS Inc., Chicago, IL).

## Results

### Allelic frequencies and genotype distributions of *MDM4* rs4245739SNP

The median age was 57 years (range, 25–82 years for Shandong set; range, 23–79 years for Jiangsu set) for the patients and 57 years (range, 19–80 years for Shandong set; range, 20–81 years for Jiangsu set) for the controls(Shandong set: *P* = 0.615; Jiangsu set: *P* = 1.000). There was no significant difference between cases and controls in sex distribution (Shandong set: 78.4% males incases vs. 76.4% in controls; *P* = 0.480; Jiangsu set: 74.0% males incases vs. 71.3% in controls; *P* = 0.479). This indicates that the frequency matching was adequate (Table 1). However, more smokers among SCLC patients were found in Shandong case-control set compared with control subjects (Shandong set: 77.8% vs. 34.2%, *P*<0.001). Similarly, there are more SCLC patients who smoking than controls in Jiangsu set (78.5% vs. 21.5%, *P*<0.001). Additionally, more patients with limited disease were observed than among ones with extensive disease in two sets (Shandong set: 56.9% vs. 43.1%; Jiangsu set: 56.5% vs. 43.5%).

**Table 1:** Distribution of selected characteristics among SCLC cases and controls.

Variable		Shandong set			Jiangsu set	
	Cases	Controls	*P* ^a^	Cases	Controls	*P* ^a^
	*n* (%)	*n* (%)		*n* (%)	*n*(%)	
	320	640		200	400	
Age (year)^b^			0.615			1.000
≤57	156(48.9)	301(47.0)		99(49.5)	198(49.5)	
>57	164(51.2)	339(53.0)		101(50.5)	202(50.5)	
Sex			0.480			0.479
Male	251(78.4)	489(76.4)		148(74.0)	285(71.3)	
Female	69(21.6)	151(23.6)		52(26.0)	115(28.7)	
Smoking status			<0.001			<0.001
Yes	249(77.8)	219(34.2)		157(78.5)	113(28.3)	
No	71(22.2)	421(65.8)		43(21.5)	287(71.8)	
Clinical stage ^c^						
Limited	182(56.9)			113(56.5)		
Extensive	138(43.1)			87(43.5)		

Note: SCLC, small cell lung cancer.

^a^Two-sided χ^2^ test.

^b^Median ages of patients for Shandong set and Jiangsu set are 57 years.

^c^Classified according to the Veterans’ Administration Lung Study Group.

As shown in Table 2, the frequency for the *MDM4* rs4245739 C allele was 0.038 and 0.073among SCLC patients and healthy controls from Shandong set, and 0.043 and 0.101 from Jiangsu set. All observed genotype frequencies in both SCLC cases and controls conform to Hardy-Weinberg equilibrium. Distributions of these *MDM4* genotypes were then compared among SCLC cases and controls. The frequencies of *MDM4* rs4245739 AA and AC or CC genotypes among patients were significantly different from those among controls in Shandong set (χ^2^ = 10.70, *P* = 0.005, *df* = 2) (Table 2). Similarly, the frequencies of *MDM4* rs4245739 AA and AC or CC genotypes among cases were significantly different from those among control subjects in Jiangsu set (χ^2^ = 12.84, *P* = 0.002, *df* = 2) (Table 2).

**Table 2:** Genotype frequencies of *MDM4*rs4245739A>C polymorphism among patients and controls and their association with SCLC risk.

Genotypes			*MDM4* rs4245739A>C		
		Cases, *n* (%)	Controls, *n* (%)	OR^a^ (95% CI)	*P*
Shandong set		*n* = 320	*n* = 640		
	AA	297(92.6)	548(85.6)	Reference	
	AC	22(7.1)	90(14.1)	0.52(0.31–0.87)	0.013
	CC	1(0.3)	2(0.3)	NC	NC
	AC+CC	23(7.4)	92(14.4)	0.53(0.32–0.89)	0.014
Jiangsu set		*n* = 200	*n* = 400		
	AA	183(91.5)	321(80.3)	Reference	
	AC	17(8.5)	77(19.3)	0.48(0.26–0.88)	0.019
	CC	0(0)	2(0.4)	NC	NC
	AC+CC	17(8.5)	79(19.7)	0.47(0.26–0.87)	0.017
Total		*n* = 520	*n* = 1040		
	AA	480(92.3)	869(83.6)	Reference	
	AC	39(7.5)	167(16.1)	0.49(0.32–0.75)	0.001
	CC	1(0.2)	4(0.4)	NC	NC
	AC+CC	40(7.7)	171(16.5)	0.50(0.32–0.76)	0.001

Note: SCLC, small cell lung cancer; NC, not calculated; OR, odds ratio; CI, confidence interval.

^a^Data were calculated by logistic regression with adjustment for age, sex, and smoking status.

### Association between *MDM4* rs4245739SNP and SCLC risk

Associations between genotypes of *MDM4* rs4245739 A>C polymorphism and SCLC risk were calculated using unconditional logistic regression analyses (Table 2). As shown in Table 2, the *MDM4* rs4245739 C allele acts as a protective allele of SCLC. Subjects with the AC genotype had an OR of 0.52 (95% CI = 0.31–0.87, *P* = 0.013) for developing SCLC, compared with subjects with the AA genotype. Significantly decreased SCLC risk was also observed among rs4245739 AC or CC carriers compared to the AA carriers (OR = 0.53, 95% CI = 0.32–0.89, *P* = 0.014). The associations of SCLC risk with the *MDM4* rs4245739SNP were further validated in an independent Jiangsu case-control set. A significantly decreased SCLC risk was also associated with rs4245739 (AC: OR = 0.48, 95% CI = 0.26–0.88, *P* = 0.019;AC or CC: OR = 0.47, 95% CI = 0.26–0.87, *P* = 0.017). In the pooled analyses, we found that the rs4245739 AC genotype had a 0.49-folddecreased risk for SCLC compared with the AA genotype (95% CI = 0.32–0.75, *P* = 0.001), and the rs4245739 AC or CC genotype had a 0.50-folddecreased risk compared with the AA genotype (95% CI = 0.32–0.76, *P* = 0.001) (Table 2). All ORs were adjusted for sex, age and smoking status.

We further examined the SCLC risk associated with the *MDM4*rs4245739genotypes by stratifying for age, sex, smoking status and disease stage using the pooled data of two Chinese case-control sets (Table 3). In stratified analyses with age, rs4245739AC and CC genotypes were significantly associated with decreased risk in subjects aged 57 years or younger (OR = 0.40, 95% CI = 0.21–0.74, *P* = 0.003), but not in subjects aged older than 57 years (OR = 0.63, 95% CI = 0.35–1.15, *P* = 0.134). There was no significant gene-age interaction (*P*_interaction_ = 0.274). A significantly decreased risk of SCLC was associated with AC and CC genotypes only among females (OR = 0.33, 95% CI = 0.15–0.74, *P* = 0.007), but not among males (OR = 0.63, 95% CI = 0.38–1.06, *P* = 0.083), compared with the *MDM4*rs4245739 AA genotype. No significant gene-sex interaction was observed (*P*_interaction_ = 0.135). We did not find significantly decreased risk (OR = 0.73, 95% CI = 0.42–1.28, *P* = 0.274) for the carriers with AC and CC genotypes compared with individuals with the AA genotype in smokers. However, thers4245739AC and CC genotypes did show a 0.30-fold decreased risk to develop SCLC (95% CI = 0.14–0.64, *P* = 0.002) compared with the AA genotype in nonsmokers. A multiplicative gene—smoking interaction existed (*P*_interactioin_ = 0.048). Associations between rs4245739 AC and CC genotypes and SCLC risk were observed in both patients with the limited stage disease (OR = 0.52, 95% CI = 0.32–0.84, *P* = 0.008) or the extensive stage disease (OR = 0.48, 95% CI = 0.24–0.97, *P* = 0.040) (Table 3).

**Table 3:** Association between *MDM4*rs4245739A>C variant andSCLC risk stratified by selected variables.

Variable		*MDM4* rs4245739A>C			*P*_interaction_ ^c^
	AA^a^	AC+CC^a^	OR^b^ (95% CI)	*P*	
Age (year)					0.274
≤57	237/396	18/103	0.40(0.21–0.74)	0.003	
>57	243/473	22/68	0.63(0.35–1.15)	0.134	
*P*_heterogeneity_	0.180	0.079			
Sex					0.135
Male	369/684	30/90	0.63(0.38–1.06)	0.083	
Female	111/185	10/81	0.33(0.15–0.74)	0.007	
*P*_heterogeneity_	0.435	0.010			
Smoking status					0.048
Nonsmoker	105/572	9/136	0.30(0.14–0.64)	0.002	
Smoker	375/297	31/35	0.73(0.42–1.28)	0.274	
*P*_heterogeneity_	<0.001	<0.001			
Stage					NC
Limited	270/869	25/171	0.52(0.32–0.84)	0.008	
Extensive	210/869	15/171	0.48(0.24–0.97)	0.040	
*P*_heterogeneity_	0.015	0.135			

Note: SCLC, small cell lung cancer; OR, odds ratio; CI, confidence interval; NC, not calculated.

^a^Number of case patients with genotype/number of control subjects with genotype.

^b^Data were calculated by logistic regression with adjustment for age, sex, and smoking status, where it was appropriate.

^c^ *P* values for gene-environment interaction were calculated using the multiplicative interaction term in SPSS software.

### Functional relevance ofrs4245739 on miRNA-mediated *MDM4* regulation

**Fig 1:**
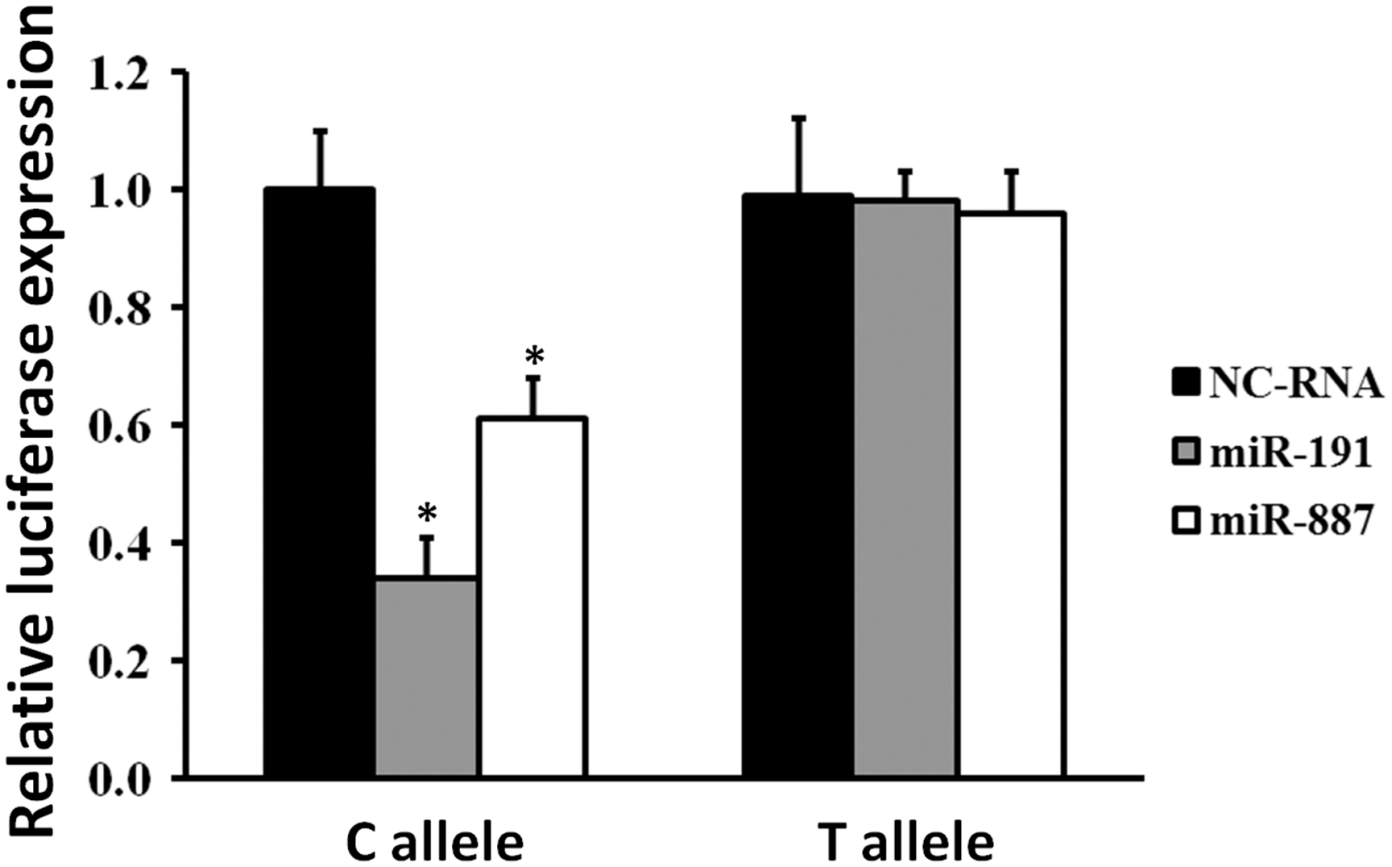
Transient luciferase reporter gene expression assays with constructs containing different alleles of *MDM4* 3’-UTR region in SCLC H446 cells. H446 cells were cotransfected with pRL-SV40 to standardize transfection efficiency. Fold increase was measured by defining the activity of cells co-transfected with both pGL3-rs4245739A or pGL3-rs4245739Creporter construct and NC-RNAs as 1. All experiments were performed in triplicates at least in three independent transfection experiments and each value represents mean ± SD. *P<0.05compared with each of the luciferase construct by *t*-tests. NC-RNA, negative control RNAs; SCLC, small cell lung cancer.

SNPrs4245739A>C could lead to higher affinity between miR-191-5p/miR-887-3p and *MDM4*mRNA and decreased *MDM4*expression in multiple malignancies [19–22]. However, its impacts on *MDM4* regulation in SCLC are still unclear. As a result, we examined whether there is an allele-specific effect of rs4245739polymorphismon *MDM4* expression in SCLC cells by miR-191-5p and miR-887-3p. Relative luciferase expression assays indicated that miR-191-5p can significant lower luciferase activities in SCLC H446 cells transfected with rs4245739C allelic reporter constructs compared to negative control RNAs(*P* = 0.003) (Fig 1). Similarly, miR-887-3p also regulates *MDM4* 3’-UTR region with significantly lower luciferase activities in H446 cells expressing rs4245739C allelic constructs compared to negative control RNAs (*P* = 0.014) (Fig 1). However, there was no such depression in H446 cells with transfection of rs4245739A allelic reporter constructs by both miRNAs mimics (Fig 1).

## Discussion

In the current study, we investigated the association between the *MDM4* rs4245739 functional SNP and SCLC risk via a case-control approach. We observed that individuals with *MDM4* rs4245739 AC or CC genotypes show significantly decreased SCLC risk compared with the AA genotype carriers. To reveal miR-191-5p/miR-887-3p mediated allelic-regulation by this SNP, we examined luciferase activities in SCLC cells transfected with different allelic reporter constructs. The genotype-phenotype correlation analyses indicated that both miRNAs could inhibit *MDM4* expression only in SCLC cells with C-allele constructs expression but not A-allele constructs. These results highlight the involvement of functional genetic variants in miRNA-binding sites in SCLC etiology.

Accumulated evidences demonstrated that genetic makeup might have direct contribution of to SCLC risk [33–35]. Interestingly, referring to the databases of gene expression profile to identify genes that are deregulated in SCLC and their SNPs in the 3’-UTR, Xiong et al identified a SCLC susceptibility SNP rs3134615 G>T can inhibit the interaction of miR-1827 with *MYCL1* 3’-UTR, resulting in higher constitutive expression of MYCL1 [35]. Consistent with this notion, we also found a *MDM4*3’-UTR SNP targeted by miRNAs is associated with SCLC susceptibility.

The *MDM4*rs4245739 polymorphism showed a consistent association with SCLC risk intwo independent case-control sets, which are unlikely to be attributable to unknown confounding factors due to having significantly increased odd ratios with small *P* values. In addition, the genotype-phenotype relationship between the rs4245739 polymorphism and reporter gene expression supports our conclusion. Moreover, the *MDM4*rs4245739 SNP might be a common genetic risk component for different cancers, which has been proved by our group and others via either candidate gene approach or GWAS [19–26]. For example,we previously found that rs4245739 AC and CC genotypes were significantly associated with decreased risk of esophageal cancer [22], a malignancy with similar environmental etiology of SCLC (i.e. heavy smoking). However, there might be several limitations in the current case-control study. For instance, since all SCLC cases were recruited from the hospital, inherent selection bias may exist. Therefore, the findings of our study warrant to be validated in a population-based prospective study in the future. Relatively small sample size for non-smokers should be further analyzed in a larger population.

In summary, our results suggested that functional *MDM4* rs4245739 SNP was associated with a significantly decreased SCLC risk in Chinese Han populations. The associations between SNPs and SCLC risk are remarkable in nonsmokers. Given this fact, further efforts are needed to examine whether *MDM4* rs4245739 genetic polymorphism can be used as a potential diagnostic marker of SCLC.
